# Statistical Modeling of Determinants of Anemia Prevalence among Children Aged 6–59 Months in Nigeria: A Cross-Sectional Study

**DOI:** 10.1155/2020/4891965

**Published:** 2020-11-03

**Authors:** Ropo Ebenezer Ogunsakin, Bayowa Teniola Babalola, Oludare Akinyemi

**Affiliations:** ^1^Discipline of Public Health Medicine (Biostatistics), University of KwaZulu Natal, Durban, South Africa; ^2^Department of Statistics, Ekiti State University, Ado Ekiti, Nigeria

## Abstract

**Objective:**

Childhood anemia remains a significant public health challenge in developing countries, and it has negative consequences on the growth of the children. Therefore, it is essential to identify the determinants of childhood anemia, as these will help in formulating appropriate health policies in order to meet the United Nations MDG goal. This study aims to assess and model the determinants of the prevalence of anemia among children aged 6–59 months in Nigeria. To accomplish the aims of the study, the authors applied single-level and multilevel binary logistic regression models.

**Methods:**

To measure the relative impact of individual and household-level factors for childhood anemia among children aged 6–59 months, this study undertakes data from Nigeria Demographic and Health Surveys with both binary logistic and multilevel logistic regression models. The fit of the model was assessed by Hosmer–Lemeshow goodness-of-fit, variance inflation factor, and likelihood ratio tests.

**Results:**

The study established that about 67.01% of the children were anemic and identified sex of children, mother's education, religion, household wealth status, total children ever born, age of children, place of residence, and region to have a statistical significant effect on the prevalence of anemia. The adjusted odds ratio (aOR) for anemia was 0.56 (95% CI = 0.50, 0.63) in children aged from 24 to 42 months and 0.40 (95% CI = 0.36, 0.45) in children aged from 43 to 59 months. Also, children who reside in certain geographical-political zones of Nigeria are associated with increased childhood anemia.

**Conclusion:**

This study has highlighted the high prevalence of childhood anemia in Nigeria and indicated the need to improve mothers' education and regional variations. Findings from this study can help policymakers and public health institutions to map out programs targeting these regions as a measure of tackling the prevalence of anemia among the Nigerian populace.

## 1. Introduction

The prevalence of anemia in children, particularly those below the age of 5 years, continues to be a significant public health challenge globally without sub-Saharan Africa (SSA). It is a severe concern for children because it can impair cognitive development and is associated with long-term health and economic consequences [1, 2]. Moreover, the consequences have a significant impact on the growth and development of children in the early stages of life. It poses a significant public health issue leading to an increased risk of child mortality. It is estimated that nearly half of all the cases of anemia are caused because of iron deficiency [3]. Unfortunately, despite all efforts made to curb the menace, anemia continues to be one of the significant and critical public health problems affecting children globally in both developing and developed countries. Based on the report from the World Health Organization (WHO) [4], it is one of the ten (10) most serious health problems globally. Approximately 273.2 million children aged 6–59 months across the world were suffering from anemia in 2011, according to a WHO report, with a prevalence rate of 42.4% [5, 6].

Furthermore, the estimated prevalence of anemia in children aged 6–59 months is 62.3% in SSA (approximately 84.5 million). These statistics put SSA as the region with the highest prevalence of anemia. Despite all the implementation of control programs such as iron supplementation and insecticide-treated bed nets distribution to curb the menace, anemia remains a major global concern in child health, particularly in SSA [7]. Meanwhile, the outcome of these community programs in SSA requires a good knowledge of the associated factors of anemia. Other studies conducted [8, 9] showed that anemia is present in 60.2–87.8% of children aged 6–59 months. A recent study found an association between anemia and some selected variables such as (age, female sex, and birth order), maternal (maternal anemia, mother's age, and mother's body mass index), and contextual (household income and family structure) characteristics of the child in children aged 6–59 months in SSA countries [10, 11].

In 2018, Nigeria Demographic Health Survey (NDHS) data revealed that the prevalence of anemia among children aged 6–59 months was also high, and anemia affected about 68.0% of these children, with 27% having mild anemia, 38% having moderate anemia, and 3% having severe anemia. Meanwhile, the WHO recommended that any prevalence of anemia that is higher than 40% among children aged 6–59 months should be considered as a severe public health problem [12]. Although many studies have examined determinants factors associated with anemia among children aged 6–59 months in Nigeria, the majority of such studies [10, 11, 13] relied on health facility-based data that are not representative considering the general population of children aged 6–59 months in the country. Also, there is information on anemia among under-five mortality prevalence in Nigeria [4]. However, limited information is reported in the literature on individual and contextual factors, especially modifiable factors that could explain the high rate of anemia in the country using nationally representative data. Because of the interrelationship of many of these factors, it is crucial to evaluate the potential determinants and assess the prevalence of anemia in a multivariable model using single-level logistic and multilevel logistic regression models. Besides, the fit of the model was examined using variance inflation factor and likelihood ratio tests. Thus, it is crucial to explore the latest Nigeria Demographic and Health Survey data that is a national representative on children aged 6–59 months by measuring the relative impact of individual and contextual determinants of the prevalence of anemia across the region of the Nigeria model. The goal is to use the findings from this study to inform and strengthen appropriate national policies and intervention strategies to reduce anemia among children in the country.

## 2. Materials and Methods

### 2.1. Study Design

This is a population-based cross-sectional study that used data obtained from the 2018 NDHS.

### 2.2. Study Population

The current study used data from the 2018 Nigeria Demographic Health Survey (NDHS) [14]. The 2018 Nigeria Demographic and Health Survey is the sixth comprehensive and national representative survey conducted in Nigeria as a part of the worldwide Demographic and Health Surveys project. The main objective of the 2018 NDHS was to provide timely and reliable data on health indicators and demographic outcomes at both national and state levels and for urban and rural areas. The sample was selected using a stratified, two-stage cluster design, with enumeration areas (EAs) as the sampling units for the first stage. The second stage was a complete listing of households carried out in each of the 1,400 selected enumeration areas (EAs). The primary sampling unit (PSU), referred to as a cluster for the 2018 NDHS, is defined based on EAs from the 2006 EA census frame. Before sample selection, all localities were classified separately into urban and rural areas based on predetermined minimum sizes of urban areas (cutoff points); consistent with the official definition in 2017, any locality with more than a minimum population size of 20,000 was classified as urban. The sample for the 2018 NDHS was a stratified sample selected in two stages. Stratification was achieved by separating each of the 36 states and the Federal Capital Territory into urban and rural areas. In total, 74 sampling strata were identified. Samples were selected independently in every stratum via a two-stage selection.

### 2.3. Ethical Consideration

This study was based on the analysis of existing survey datasets in the public domain that are available free online. The first author obtained permission for the download and usage of the NDHS datasets from http://www.dhsprogram.com/data/dataset_admin/login_main.cfm.

### 2.4. Outcome of Interest

Based on previous studies, hemoglobin concentration has been considered as the most reliable indicator of anemia at the population level. As the standard of the WHO, children aged 6–59 months with a hemoglobin level of less than 11 g/dL are declared as anemic. A drop of blood from the prick site was drawn into a microcuvette, and a hemoglobin analysis was carried out on-site with a battery-operated portable HemoCue analyzer to measure the prevalence of low hemoglobin [14, 15]. Besides, parents of children with a hemoglobin level below 11 g/dl were directed to take the child to a health facility for follow-up care. Based on hemoglobin levels, the prevalence of anemia is adjusted for altitude by hemoglobin in grams per deciliter (g/dl) [15]. In the present study, the outcome variable (anemia status) was dichotomized, suggesting whether one is anemic or not and is categorized as being anemic (coded as 1) or nonanemic (coded as 0).

(1) $$\begin{array}{c} y_{i}=\left\{\begin{array}{c} 1\text{ haemoglobin}<12.0\;\text{g}/\text{dL}\left(\text{anemic}\right),\\ 0\text{ haemoglobin}\geq 12.0\;\text{g}/\text{dL}\left(\text{non-anemic}\right). \end{array}\right. \end{array}$$

### 2.5. Description of the Explanatory Variables

The covariates considered include both individual and community-level factors as predictors of anemia among children aged 6–59 months. Among the selected explanatory variables, some were related to the child such as sex of the child, age (divided into three categories: 6–23 months, 24–42 months, and 43–59 months), anemia status, type of birth, and size of the child at birth. Other variables related to household-level factors included in the analysis are education, breastfeeding, the current age of the respondent, total children ever born, number of children under-five in the household, number of births in the last five years, religion, and wealth index. The wealth index is a proxy indicator of the socioeconomic status derived based on the scores allocated to various household items. Thus, the total scores were then grouped into wealth quintiles: most inferior, lower, middle, richer, and richest. The study also adjusts for potential confounders termed as community-level factors that include ethnicity, the region of the respondent, and place of residence.

The first inclusion criterion was that the child must be aged 6–59 months. Variables considered in this study were selected based on literature that has been conducted at the global level. Potential determinant factors expected to be correlated with anemia status were included as variables of the study. The exclusion criteria were the variables that have not been identified as modifiable factors in the literature. Besides, any variables with missing value > 80% were excluded from this study.

### 2.6. Statistical Analysis

Descriptive statistics of each of the selected variables and distribution of anemia by different factors were presented. Further analyses were conducted to examine child, individual, and community-level factors that might be significantly associated with childhood anemia and explored unobserved household-level effects on childhood anemia. Based on a previous study that utilized the type of data under investigation, a single-level model would not be sufficient for removing the cluster effect in hierarchical structure data. However, the hierarchical structure sometimes yields highly correlated data and thus cannot be assumed independent. A multilevel approach adequately represents the unexplained variability of the nested structure, often difficult to present in a single-level approach. Thus, the current study extends the best model from single-level binary logistic to multilevel logistic regression modeling in order to take into consideration the hierarchical structure and the possible correlation that may arise [16, 17]. Besides, the multilevel logistic regression model is a powerful statistical tool for removing the cluster effect and detecting associations between the outcome of interest and the explanatory variables at different levels of the data hierarchy. This model is particularly appropriate for research designs where participants are organized at more than one level [18].

To check for multicollinearity, some studies choose to use generalized variance inflation (GVIF) factors or variance inflation factors (VIF). According to [19, 20], if all terms in an unweighted linear model have one degree of freedom (DF), then the usual variance inflation factors are calculated; otherwise, generalized variance inflation factors are preferred. Based on this, the current study only investigates the VIF of all the covariates included in the model. The variance inflation factor represents the proportion of variance in one predictor explained by all the other explanatory variables in the model. The approach suggested by [21] is to calculate VIFs for each parameter in the model, and if they are larger than a cutoff, sequentially drop the covariate with the largest VIF, recalculate, and repeat until all values are below the cutoff (they suggested a cutoff of 2). VIFs are especially suitable for dealing with the collinearity of interaction terms. As a rule of thumb, a VIF value that exceeds 5 or 10 indicates a problematic amount of collinearity [22, 23]. Data management and cleaning were carried out using IBM Statistical Package for Social Sciences (SPSS) Statistics for Windows version 26.0. We fitted the model using the *R* software (R Core Team, Vienna, Austria) [24].

## 3. Results

### 3.1. Sample Characteristics

The details of the descriptive statistics are presented in Table 1. About 67.01% of the children was anemic. The children were relatively evenly distributed in terms of sex. The mean (SD) of the current respondent age was 30.00 ± 6.69, while that of the child age (in the month) was 31.46 ± 15.64. Out of 10,451 children aged 6–59 months in the dataset, 10,125 were born singletons and 284 (2.72%) were born very small (born underweight). The proportion of children belonging to the household with no formal education and higher education is 38.45% and 9.01%, respectively. The majority (52.48%) of the children belonged to women in Islamic religion, while 211 (20.20%) belonged to households with the poorest wealth index. About 60.94% of the children resides in rural communities, and the majority (59.63%) reside in the northern region of the country, of which northwest has the highest proportion (24.31%) (Table 1 for each covariate). The bivariate association between the anemia status of children aged 6–59 months and covariates was also assessed, and the results are shown in (Table 2). The results highlighted the significant determinants of variation in the prevalence of anemia among children aged between 6 and 59 months. Birth type and sex of household were the only explanatory variables that had no significant association with anemia status among children aged 6–59 months.

From Table 3, the analysis of single-level binary logistic regression revealed that the prevalence of anemia among children aged between 6 and 59 months varied among different explanatory variables. Besides, the total children ever born, sex of children, mother's education, religion, household wealth status, age of children, place of residence, and region were also significant determinants of variation in the prevalence of anemia among children aged between 6 and 59 months. In contrast, the source of drinking water, currently breastfeeding, size of the child at birth, and ethnicity were insignificant predictors of variation in the prevalence of anemia among children aged between 6 and 59 months. Altogether, we fitted three (3) models, and model 3 provided the best fit to the data. Among the three models, the findings reveal model 3 to be the best fit to the data. Based on the explanation given under the statistical analysis section, we further extend model 3 to a multilevel (mixed effect) logistic regression model (model 4) to explore any unobserved household-level effects on childhood anemia. Comparing models 3 and 4, model 3 is preferred. Thus, no significant unobserved household-level variations were observed in anemia prevalence outcome after adjusting for the other explanatory variables in the model (Table 4).

Furthermore, the total children ever born (aOR = 1.02 (95% CI =  1.00, 1.04)) is associated with an increase in childhood anemia. Before adjusting for all other covariates, being a reduced Christian odds of childhood anemia by 4%, it is not significantly associated with the anemia. Wealth status was found to have protective effects on childhood anemia before adjusting for all other factors. The likelihood of childhood anemia in children from the richest household was reduced by 58% compared to children from the poorest households (Table 3, model 2). Female children have decreased odds of childhood anemia compared to male children (aOR = 0.84 (95% CI =  0.77, 1.92)). The findings of this study also indicate that the child's late age and his mother's higher-level education were negatively associated with childhood anemia. The adjusted odds ratio (aOR) for anemia was 0.56 (95% CI = 0.50, 0.63) in children aged from 24 to 42 months and 0.40 (95% CI = 0.36, 0.45) in children aged from 43 to 59 months. Concerning mother's education, we observed that primary level education was positively associated with childhood anemia (aOR = 0.89 (95% CI = 0.76, 1.02)). Moreover, the odds of childhood anemia in rural areas increased by 14% compared to urban areas. On the other hand, odds of childhood anemia were noticed to vary across the regions; in southeast and southwest, it was observed to increase by 87% and 16%, respectively, but reduced by 31% in the northwest compared to northcentral (Table 3, model 3).

### 3.2. Model Fit and Selection

The fitness of the models was examined by employing the likelihood ratio test (deviance, AIC, and BIC Values) and Hosmer–Lemeshow goodness-of-fit test, and the findings indicate that the models were fitted well as indicated in Table 5. The likelihood ratio test (deviance, AIC, and BIC values) suggests that model 3 in Table 5 provides an excellent fit to the data. For the multicollinearity, the VIF was employed to check for multicollinearity, none of the VIF values was up to 10, and the mean VIF of the model was less than six (6). This implies that there was no collinearity in the model.

## 4. Discussion

This study models the determinants of the prevalence of anemia among children aged 6–59 months using the 2018 Nigeria Demographic and Health Survey (NDHS).

Furthermore, we also examined unobserved household-level variations in childhood anemia that represents differences in childhood anemia outcomes across households. The present study has attempted to control for the effects of potential confounders by incorporating many explanatory factors into the analyses without overfitting the model. This study established a mother's education, religion, household wealth status, total children ever born, and age of children as the significant determinants of variation in the prevalence of anemia among children between 6 and 59 months. Confounding factors associated with childhood anemia were the sex of the child, ethnicity, place of residence, and region. The findings of this study also showed a high prevalence of anemia among Nigeria children aged from 6 to 59 months, making this a serious public health issue. The results also showed regional variations in the prevalence of childhood anemia among children aged 6–59 months, with the highest rate being observed in the Northern region of the country. More than half (67.01%) of the children studied were anemic, and the WHO considers anemia in children aged 6–59 months as a severe public health problem [4, 12]. However, more importantly, in this study is the unobserved household-level variations on childhood anemia.

In this study, we found anemia to be associated with the characteristics of the child and those of his mother. A possible explanation could be that children who are getting older receive a balanced diet that is richer and complete, with a sufficient intake of iron that could prevent the occurrence of anemia in the child. This finding is consistent with other previous studies in the literature [25, 26].

This study revealed that the mother's education level was negatively associated with childhood anemia except those with primary education. Notably, children whose mothers had a secondary and higher education were less likely to be anemic. A reduction in the likelihood of anemic among children from mothers with higher educational status compared to children from mothers without formal education suggests that improving maternal education will improve the likelihood of children being anemic, which is consistent with other previous studies [25, 27–29]. It is an occurrence phenomenon that improvements in the educational level of women would bring several advantages to their children and the community, as reported in the previous studies [30, 31]. A likely explanation for this is that mothers with higher educational status are more likely to provide a healthy and hygienic balanced diet, resulting in better health outcomes for both mothers and their children. It is essential to mention that this study also reveals that the likelihood of childhood anemia increases with total children ever born and the number of mothers currently breastfeeding. These findings were consistent with other previous studies [32–35]. An increment in total children ever born and currently breastfeeding could result in a lack of adequate care, low birth weight, premature births, and massive drain on the limited household resources. The implication is that children have to compete for the little resources available for their survival. The findings of this study also show that children raised in urban areas were less likely to be anemic than those in rural areas. This finding supported what has been reported in other studies [8, 10, 36]. A possible explanation for this relationship is that children in urban areas have better access to health facilities and other essential health-related services that are important in developing the children in the early stages of life.

Children residing in the southeast region have an increased risk of childhood anemia compared to children living in the Western region. The rate is highest in the northern, followed by the southeast. This study is consistent with previous findings that geographical locations of children influence their health outcomes [11, 16, 18–20]. It is essential to mention that the distribution of socioeconomic resources and political power largely influences the health conditions of populations at the local and regional levels across Nigeria.

Also, the regional differences in childhood anemia in the country could be attributed to the variations in the implementation of national health policies and inadequate health services and poor living conditions of households. The wealth status of richer households is negatively associated with childhood anemia. The findings of this study reveal that children from less wealthy households have greater odds of being anemic than children from wealthy households. Similar results were documented in previous studies; our study gives further proof that poverty is a significant determinant of childhood anemia [37–42]. It is essential to mention that the level of socioeconomic status of a household is crucial. It often determines the availability of adequate and nutritious foods for the growth and development of children. For example, children from poorer households in most developing countries, such as Nigeria, where a publicly funded health care system lacks access to excellent and necessary infrastructural healthcare services may anytime fall ill. Additionally, childhood anemia is an urgent public health problem in Nigeria, and this study has provided vital information for understanding and addressing anemia in the country. Policies and intervention strategies targeted at improving the level of mother education could play a crucial role in the health status of children if supported with efforts to improve the general standard of living among households in the entire country.

## 5. Strengths and Limitations

The strengths of the study lie on the fact that the data are a national representative population-based study coupled with useful quality data on children's health, their households, and communities. Besides, the study also has a large sample size drawn randomly nationwide, making it possible to generalize findings on children aged 6–59 months. The multilevel logistic modeling approach also allowed the determination of unobserved household-level differences in the prevalence of anemia, which cannot be determined through single-level binary logistic regression. The main limitation of this study stems from the fact that the demographic health survey datasets are prone to problems that result in the inability to measure causal effects due to the cross-sectional nature.

## 6. Conclusion

Summarily, results have highlighted a high prevalence of childhood anemia in Nigeria. The policymakers should pay attention to all the statistically significant factors outlined in the analysis of the current study. In light of the significant associated factors of anemia prevalence, a pragmatic approach is required from the policymakers. This would help in addressing the menace of childhood anemia in response to the growth and development of the children in the early stages of life, particularly for children who are living in the highest anemic prevalence regions.

## Figures and Tables

**Table 1:** Individual-level, community-level, and household-related characteristics of the study participants (*n* = 10,451).

Variables		Frequency (*n*)	Percentage
Respondent age: 30.00 ± 6.69			
*Individual-level factors*			
Sex of the child	Male	5288	50.6
	Female	5163	49.4
Child age in month	6–23	3712	35.53
	24–41	3229	30.90
	42–59	144	1.38
Birth type	1^st^ of multiple	182	1.74
	2^nd^ of multiple	144	1.38

*Community-level factors*			
Type of residence	Urban	4082	39.06
	Rural	6369	60.94
Geographical area	Northcentral	1808	17.30
	Northeast	1883	18.02
	Northwest	2541	24.31
	Southeast	1527	14.61
	South-south	1179	11.28
	Southwest	1513	14.48

*Household factors*			
Educational status	No formal education	4018	38.45
	Primary	1763	16.87
	Secondary school	3728	35.67
	Higher education	942	9.01
Source of drinking water	Improved	6504	62.23
	Unimproved	3947	37.77
Religion	Catholic	1069	10.23
	Christian	3799	36.35
	Islamic	5485	52.48
	Traditionalist	98	0.41
	No religion	55	0.53
Currently breastfeeding	No	5305	50.76
	Yes	5146	49.24

*Household factors*			
Wealth index	Poorest	2111	20.20
	Poorer	2075	19.85
	Middle	2300	22.01
	Richer	1780	17.03
	Richest	6707	22.0
Size of child at birth	Very large	996	9.53
	Larger than average	2542	24.32
	Average	5531	52.92
	Smaller than average	957	9.16
	Very small	284	2.72
	Do not know	141	1.35

**Table 2:** Prevalence of anemia by various explanatory factors.

Covariates	Categories	Not anemic	Anemic	*P* value
*Individual-level factors*				
Sex of child	Male	1650 (31.20)	3638 (68.80)	<0.0001
	Female	1798 (32.82)	3365 (65.18)	
Child age (in month)	6–23	866 (23.33)	2846 (76.67)	0.0261
	24–41	1229 (35.01)	2281 (64.90)	
	42–59	1353 (41.90)	1876 (58.10)	
Birth type	Single birth	3351 (33.10)	6774 (66.90)	0.4092
	1^st^ of multiple	56 (30.77)	126 (69.23)	
	2^nd^ of multiple	41 (28.47)	103 (71.53)	

*Household level factors*				
Educational level	No formal education	1063 (26.46)	2955 (73.54)	<0.0001
	Primary	532 (30.18)	1231 (69.82)	
	Secondary	1356 (36.37)	2372 (63.63)	
	Tertiary	497 (52.76)	445 (47.24)	
Source of drinking water	Improved	2255 (34.67)	4249 (65.33)	<0.0001
	Unimproved	1193 (30.23)	2754 (69.77)	
Wealth index	Poorest	487 (23.07)	1624 (76.93)	<0.0001
	Poorer	537 (25.88)	1538 (74.12)	
	Middle	780 (33.91)	1520 (66.09)	
	Richer	781 (35.74)	1404 (64.26)	
	Richest	863 (48.48)	917 (51.52)	
Currently breastfeeding	No	1946 (36.68)	3359 (63.32)	<0.0001
	Yes	1502 (29.19)	3644 (70.81)	
	Don't know	47 (33.33)	94 (66.67)	
Sex of household	Male	3012 (32.75)	6186 (67.25)	0.148
	Female	436 (34.80)	817 (65.20)	
Size of child at birth	Very large	334 (33.53)	662 (66.47)	0.045
	Larger than average	821 (32.30)	1721 (67.70)	
	Average	1883 (34.04)	3648 (65.96)	
	Smaller than average	280 (29.26)	677 (70.74)	
	Very small	83 (29.23)	201 (70.77)	
	Don't know	47 (33.33)	94 (66.67)	

*Communitylevel factors*				
Place of residence	Urban	1613 (39.51)	2469 (60.49)	<0.0001
	Rural	1835 (28.81)	4534 (71.19)	
Region	North-central	632 (34.96)	1776 (65.04)	<0.0001
	North-east	610 (32.40)	1273 (67.60)	
	North-west	760 (29.91)	1781 (70.09)	
	South-east	477 (31.24)	1050 (68.76)	
	South-south	351 (29.77)	828 (70.23)	
	South-west	618 (40.85)	895 (59.15)	

**Table 3:** Estimates of multiple logistic regression models (NDHS 2018).

Covariates	Model 1	Model 2	Model 3
	aOR (95% CI)	aOR (95% CI)	aOR (95% CI)
Sex of child (ref: male)			
Female	0.85 (0.78, 0.92)^*∗*^		0.84(0.77, 0.92)^*∗*^
Type of birth (ref: single)			
1^st^ of multiple	1.11 (0.80, 1.52)		1.15 (0.83, 1.62)
2^nd^ of multiple	1.27 (0.88, 1.82)		1.42 (0.97, 2.09)
Education (ref: no formal education)			
Primary		0.97 (0.85, 1.12)^*∗*^	0.89 (0.76, 1.02)^*∗*^
Secondary		0.82 (0.72, 0.95)^*∗*^	0.75 (0.65, 0.87)^*∗*^
Tertiary		0.53 (0.43, 0.64)	0.51 (0.41, 0.62)
Source of drinking water (ref: improved)			
Unimproved		0.99 (0.90, 1.10)	0.98 (0.89, 1.09)
Religion (ref: Catholic)			
Christian		1.04 (0.90, 1.21)	1.16 (0.99, 1.37)
Islamic		0.92 (0.79, 1.08)	1.55 (1.28, 1.86)
Traditionalist		0.49 (0.26, 0.92)^*∗*^	0.61 (0.32, 1.18)
no religion		1.35 (0.74, 2.46)	1.19 (0.62, 2.26)
Wealth index (ref: poorest)			
Poorer		0.88 (0.76, 1.02)	0.86 (0.74, 1.01)
Middle		0.62 (0.54, 0.73)8	0.58 (0.49, 0.68)^*∗*^
Richer		0.61 (0.53, 0.73)^*∗*^	0.55 (0.46, 0.65)^*∗*^
Richest		0.42 (0.35, 0.50)^*∗*^	0.38 (0.31, 0.46)^*∗*^
Currently breastfeeding (ref: no)			
Yes		1.05 (0.96, 1.15)	1.07 (0.97, 1.17)^*∗*^
Total children ever born		1.02 (1.00, 1.04)^*∗*^	1.02 (1.00, 1.04)^*∗*^
Size of child at birth (ref: very large)			
Larger than average		0.99 (0.84, 1.16)	0.95 (0.81, 1.13)
Average		0.93 (0.80, 1.08)	0.88 (0.76, 1.02)
Smaller than average		1.06 (0.88, 1.30)	1.02 (0.83, 1.25)
Very small		1.00 (0.75, 1.35)	0.92 (0.68, 1.24)
Do not know		1.03 (0.70, 1.52)	0.89 (0.60, 1.32)
Child age in month (ref: 6–23)			
24–41		0.56 (0.50, 0.63)^*∗*^	0.56 (0.50, 0.63)
42–59		0.41 (0.36, 0.45)^*∗*^	0.40 (0.36, 0.45)^*∗*^
Ethnicity (ref: ekoi)			
Fulani			0.91 (0.70, 1.18)^*∗*^
Hausa			0.99 (0.74, 1.32)
Igbo			0.95 (0.83, 1.11)
Yoruba			0.96 (0.76, 1.23)
Place of residence (ref: urban)			
Rural			1.14 (1.02, 1.27)^*∗*^
Region (ref: north central)			
Northeast			0.76 (0.63, 0.89)^*∗*^
Northwest			1.87 (1.41, 2.49)^*∗*^
Southeast			0.87 (1.54, 2.28)^*∗*^
South-south			1.19 (0.62, 2.26)^*∗*^
Southwest			1.16 (0.94, 1.43)

^*∗*^ *p* < 0.05.

**Table 4:** Estimates of multilevel logistic regression model (NDHS 2018).

Covariates	aOR (95% CI)
Sex of child (ref: male)	—
Female	0.96 (0.94, 0.98)^*∗*^
Type of birth (ref: single)	
1^st^ of multiple	1.03 (0.07, 1.107)
2^nd^ of multiple	1.07 (1.00, 1.16)^*∗*^
Education (ref: no formal education)	—
Primary	0.97 (0.94, 1.01)
Secondary	0.94 (0.91, 0.97)^*∗*^
Tertiary	0.86 (0.82, 0.90)
Source of drinking water (ref: improved)	—
Unimproved	1.00 (0.98, 1.02)
Religion (ref: catholic)	—
Christian	1.02 (0.98, 1.06)
Islamic	1.08 (1.04, 1.13)^*∗*^
Traditionalist	0.89 (0.77, 1.03)
No religion	1.07 (0.93, 1.23)
Wealth index (ref: poorest)	—
Poorer	0.97 (0.94, 1.01)
Middle	0.91 (0.94, 1.01)^*∗*^
Richer	0.89 (0.86, 0.93)^*∗*^
Richest	0.83 (0.79, 0.87)^*∗*^
Currently breastfeeding (ref: no)	—
Yes	1.01 (0.98, 1.03)
Total children born	1.00 (0.98, 1.03)
Size of child at birth (ref: very large)	—
Larger than average	0.99 (0.95, 1.02)
Average	0.97 (0.94, 1.01)
Smaller than average	1.00 (0.96, 1.04)
Very small	0.98 (0.92, 1.05)
Don't know	0.97 (0.89, 1.05)
Child age in month (ref: 6–23)	—
24–41	0.89 (0.87, 0.91)^*∗*^
42–59	0.83 (0.81, 0.85)^*∗*^
Ethnicity (ref: ekoi)	—
Fulani	0.99 (0.93, 1.05)
Hausa	0.99 (0.93, 1.06)
Igbo	0.94 (0.91, 1.03)
Yoruba	0.99 (0.94, 1.05)
Place of residence (ref: urban)	—
Rural	1.04 (1.01, 1.07)^*∗*^
Region (ref: north central)	—
North-east	0.93 (0.90, 0.98)^*∗*^
North-west	0.96 (0.92, 1.00)
South-east	1.14 (1.07, 1.22)^*∗*^
South-south	1.13 (1.08, 1.19)^*∗*^
South-west	1.04 (0.98, 1.09)

**Table 5:** Goodness-of-fit assessment for both single-level logistic and multilevel logistic models using AIC, BIC, and deviance.

Parameters	Model 1	Model 2	Model 3	Model 4
AIC	13244.90	12598.00	12470.60	13173.89
BIC	13273.92	12757.60	12724.51	13442.31
Deviance	13236.90	12554.00	12400.60	13099.89

## Data Availability

The secondary data used in this analysis are available upon request to the Nigeria Demographic Health Survey (website:http://www.dhsprogram.com/data/dataset_admin/login_main.cfm). The first author took permission to download the dataset from their website. Hence, the data used to support the findings of this study are available from the corresponding author upon request.

## References

[1] Crawley J. (2004). Reducing the Burden of anemia in infants and young children in malaria-endemic countries of africa: from evidence to action. *The American Journal of Tropical Medicine and Hygiene*.

[2] Balarajan Y., Ramakrishnan U., zaltin E. O., Anuraj H., Subrama- nian S. V. (2011). Anaemia in low-income and middle-income countries. *The Lancet*.

[3] Soleimani N. (2011). Relationship between anaemia, caused from the iron deficiency, and academic achievement among third grade high school female students. *Procedia-Social and Behavioral Sciences*.

[4] Sanou D., Turgeon-O’Brien H., Desrosiers T. (2008). Prévalence et déterminants non alimentaires de l’anémie et de la carence en fer chez des orphelins et enfants vulnérables d’âge préscolaire du Burkina-Faso. *Nutrition Clinique et Métabolisme*.

[5] Geneva S. (2011). Haemoglobin concentrations for the diagnosis of anaemia and assessment of severity.

[6] Stevens G. A., Finucane M. M., De-Regil M. (2013). Global, regional, and national trends in haemoglobin concentration and prevalence of total and severe anaemia in children and pregnant and non-pregnant women for 1995–2011: a systematic analysis of population- representative data. *The Lancet Global Health*.

[7] Kassebaum N. J., Jasrasaria R., Naghavi M. (2014). A systematic analysis of global anemia burden from 1990 to 2010. *Blood*.

[8] Ughasoro M. D., Emodi I. J., Hu O., Ibe B. C. (2015). Prevalence and risk factors of anaemia in paediatric patients in south-east Nigeria. *South African Journal of Child Health*.

[9] Onyemaobi G. A., Onimawo I. A. (2011). Risk factors for iron deficiency anaemia in under-five children in imo state, Nigeria. *Journal of Applied Sciences Research*.

[10] Moschovis P. P., Wiens M. O., Arlington L. (2018). Individual, maternal and household risk factors for anaemia among young children in sub-saharan africa: a cross-sectional study. *BMJ Open*.

[11] Diouf S., Folquet M., Mbofung K. (2015). Prévalence et déterminants de l’anémie chez le jeune enfant en Afrique francophone - implication de la carence en fer. *Archives de Pédiatrie*.

[12] World Health Organization (2015). *The Global Prevalence of Anaemia in 2011*.

[13] Gayawan E., Arogundade E. D., Adebayo S. B. (2014). Possible determinants and spatial patterns of anaemia among young children in Nigeria: a Bayesian semi-parametric modelling. *International Health*.

[14] National Population Commission (2019). *Nigeria Demographic and Health Survey 2018: Key Indicators Report*.

[15] Centers for Disease Control (1998). Prevention Recommendations to prevent and control iron deficiency in the United States. *MMWR RR*.

[16] Aheto J. M. K., Taylor B. M., Keegan T. J., Diggle P. J. (2017). Modelling and forecasting spatio-temporal variation in the risk of chronic malnutrition among under-five children in Ghana. *Spatial and Spatio-Temporal Epidemiology*.

[17] Tom A. B., Bosker R. J. (1999). An introduction to basic and advanced multilevel modeling. *Journal of the American Statistical Association*.

[18] Khan H. R., Shaw E. (2011). Multilevel logistic regression analysis applied to binary contraceptive prevalence data. *Journal of Data Science*.

[19] Fox J., Monette G. (1992). Generalized collinearity diagnostics. *Journal of the American Statistical Association*.

[20] Fox J. (1997). *Applied Regression Analysis, Linear Models, and Related Methods*.

[21] Zuur A. F., Ieno E. N., Elphick C. S. (2010). A protocol for data exploration to avoid common statistical problems. *Methods in Ecology and Evolution*.

[22] Bruce P., Bruce A. (2017). *Practical Statistics for Data Scientists: 50 Essential Concepts*.

[23] James G., Witten D., Hastie T., Tibshirani R. (2013). *An introduction to Statistical Learning*.

[24] R Core Team (2015). *R: A Language and Environment for Statistical Computing*.

[25] PLoS One Staff (2015). Correction: prevalence and risk factors of anaemia among children aged between 6 months and 14 years in Kenya. *PloS One*.

[26] Magalhaes R. J. S., Archie C. A. (2011). Mapping the risk of anaemia in preschool-age children: the contribution of malnutrition, malaria, and helminth infections in west africa. *PLoS Medicine*.

[27] Hazavehei S. M. M., Jalili Z., Heydarnia A. R., Faghihzadeh S. (2006). Application of the precede model for controlling iron-deficiency anemia among children aged 1–5, Kerman, Iran. *Promotion & Education*.

[28] Nisar N., White F. (2008). Factors affecting utilization of antenatal care among reproductive age group women (15–49 years) in an urban squatter settlement of Karachi. *Journal of Pakistan Medical Association*.

[29] Turyashemererwa F. M., Kikafunda J., Annan R., Tumuhimbise G. A. (2013). Dietary patterns, anthropometric status, prevalence and risk factors for anaemia among school children aged 5-11 years in Central Uganda. *Journal of Human Nutrition and Dietetics*.

[30] Madise N. J., Mpoma M. (1997). Child malnutrition and feeding practices in Malawi. *Food and Nutrition Bulletin*.

[31] Adekanmbi V. T., Kayode G. A., Uthman O. A. (2013). Individual and contextual factors associated with childhood stunting in Nigeria: a multilevel analysis. *Maternal & Child Nutrition*.

[32] Kayode G. A., Adekanmbi V. T., Uthman O. A. (2012). Risk factors and a predictive model for under-five mortality in Nigeria: evidence from Nigeria demographic and health survey. *BMC Pregnancy and Childbirth*.

[33] McElroy P., Kuile F. O., Altaf A Lal P. B. B. (2000). Effect of plas-modium falciparum parasitemia density on hemoglobin concentrations among full-term, normal birth weight children in western Kenya. *The American Journal of Tropical Medicine and Hygiene*.

[34] Walker S. P., Wachs T. D., Grantham-McGregor S. (2011). Inequality in early childhood: risk and protective factors for early child development. *The Lancet*.

[35] Walker S. P., Wachs T. D., Meeks Gardner J. (2007). Child development: risk factors for adverse outcomes in developing countries. *The Lancet*.

[36] Nambiema A., Robert A., Yaya I. (2019). Prevalence and risk factors of anemia in children aged from 6 to 59 months in Togo: analysis from Togo demographic and health survey data, 2013–2014. *BMC Public Health*.

[37] E Agho K., Dibley M. J., D’Este C., Gibberd R. (2008). Factors associated with haemoglobin concentration among timor-leste children aged 6–59 months. *Journal of Health, Population, and Nutrition*.

[38] Alene K. A., Mohamed Dohe A. (2014). Prevalence of anemia and associated factors among pregnant women in an urban area of eastern Ethiopia. *Anemia*.

[39] Yaya S., Ekholuenetale M., Tudeme G., Shah V., Bishwajit G., Bernard K. (2017). Prevalence and determinants of childhood mortality in Nigeria. *BMC Public Health*.

[40] Abir T., Agho K. E., Page A. N., John Dibley M. (2015). Risk factors for under-5 mortality: evidence from Bangladesh demo- graphic and health survey, 2004–2011. *BMJ Open*.

[41] Odunayo S. I., Oyewole A. O. (2006). Risk factors for malnutri- tion among rural nigerian children. *Asia Pacific Journal of Clinical Nutrition*.

[42] Hien N. N., Kam S. (2008). Nutritional status and the characteristics related to mal- nutrition in children under five years of age in nghean, vietnam. *Journal of Preventive Medicine and Public Health*.

